# Climate-smart radiography in Ghana: training needs of diagnostic radiographers mapped to the WHO operational framework and UNFCCC Action for Climate Empowerment (ACE)

**DOI:** 10.1186/s12913-025-13881-5

**Published:** 2026-01-07

**Authors:** Christian Ven Emery, Eric Akpabli, Bernard Amedzoame, Tretu Beracah, Jeffery Gameli Amlalo, Hayford Insidey, Isaac Tigbee, Joseph Amihere Ackah, Wuni Abdul-Razak

**Affiliations:** 1 School of Life and Health Sciences, Birmingham City University, Birmingham, UK; 2 Department of Nuclear Safety and Security, University of Ghana, Accra, Ghana; 3 Medical Physics Department, School of Nuclear and Allied Sciences, University of Ghana, Atomic Campus, Accra, Ghana; 4 Klintaps University College of Health and Allied Sciences, Tema, Ghana; 5 Richard Novati Catholic Hospital, Sogakope, Volta Region Ghana; 6 Department of Imaging Technology and Sonography, School of Allied Health Sciences, University of Cape Coast, Cape Coast, Ghana; 7 Delft Imaging, Accra, Ghana; 8 Department of Medical Imaging, University for Development Studies, Tamale, Ghana; 9 Department of Health Technology and Informatics, the Hong Kong Polytechnic, University, Kowloon, Hong Kong SAR China; 10 Fatima College of Health Sciences, Al Ain, UAE

**Keywords:** Health workforce, Diagnostic radiographers, Sustainability training, WHO operational framework, UNFCCC Action for Climate Empowerment

## Abstract

**Introduction:**

Medical imaging contributes approximately 1% of global carbon emissions and 10% of healthcare waste. Diagnostic radiographers influence this impact through decisions related to energy use, waste generation, and sustainable practice adoption. This study examined the training and educational needs of diagnostic radiographers in Ghana regarding sustainable radiography and mapped these needs against the WHO Operational Framework (Component 2: Climate-Smart Health Workforce) and the UNFCCC Action for Climate Empowerment (ACE) pillars.

**Methods:**

A descriptive cross-sectional online survey was conducted among 126 diagnostic radiographers in Ghana between December 2024 and April 2025, representing 72% of the calculated target sample size of 175. Participants completed a self-administered questionnaire via Google Forms, coordinated through the Ghana Society of Radiographers. Descriptive statistics and Spearman’s rank correlations (*p* < .05) were performed. The findings were interpreted as post-hoc using the WHO and ACE frameworks to identify alignment with global sustainability competencies.

**Results:**

Most participants (65.9%) were at least somewhat familiar with sustainability concepts, and 85.0% considered them highly relevant. However, only 35.7% felt very confident in eco-friendly practices. Nearly 40% had received no prior training, and over half reported no workplace programmes. Priority training areas included reducing energy consumption (75.4%), developing eco-friendly protocols (73.0%), and waste management (67.5%). Key barriers were lack of resources and funding (68.3%), limited knowledge (62.7%), and limited management support (61.9%). Familiarity correlated positively with confidence (ρ = 0.319, *p* < .001) and negatively with perceived knowledge gaps.

**Conclusion:**

Radiographers in Ghana recognize the importance of sustainable radiography but face knowledge, training, and resource limitations that restrict implementation. Developing targeted, cost-effective, and context-specific training that are aligned with the WHO Operational Framework and UNFCCC ACE pillars is essential to strengthen capacity for climate-smart radiography in Ghana and comparable lower-middle-income countries.

**Supplementary Information:**

The online version contains supplementary material available at 10.1186/s12913-025-13881-5.

## Introduction

Climate change, driven by rising carbon emissions and global warming, disrupts ecosystems and health systems across the globe [1]. The Intergovernmental Panel on Climate Change forecasts that temperature rises of 1.5 °C to 2 °C will severely impact life on Earth, where the harshest effects are expected in lower- and middle-income countries (LMICs) [2, 3]. Global temperatures have increased by 1 °C since the medieval era, and with the sharpest rise occurring in the past four decades [4]. Although the greenhouse effect is essential for sustaining life, excess accumulation of these greenhouse gases unsettle climate systems and poses serious health risks [5].

Healthcare generates around four million tonnes of waste annually around the world, which increases environmental pollution and depletes ecological resources [6–8]. These emissions result from both direct clinical activity and upstream supply [9, 10]. The US alone produces nearly one-quarter of the world’s greenhouse gas emissions from healthcare [11–13]. Diagnostic radiography and radiotherapy are among the most energy-intensive sectors in healthcare, which contribute approximately 1% of all global carbon emissions and 10% of healthcare waste [3, 14–16]. These emissions originate from the high-energy consuming imaging modalities, waste from single-use materials, radiopharmaceuticals, travel, and data storage [17–19]. Modalities such as MRI, CT, and SPECT are the most energy-intensive [20, 21], with MRI systems requiring constant energy supply even when idle [16, 22].

Diagnostic radiographers are frontline decision-makers who are well-positioned to influence these outcomes. Their decisions directly affect imaging resource use, patient throughput, and equipment energy consumption [23–25]. Several radiographers have already adopted sustainable practices such as switching off equipment, using appropriate contrast doses, avoiding printed reports, holding virtual meetings, and relying on digital workflows [16, 25–28]. However, the change in individual behaviour is not enough, system-level change is needed. Building a sustainable imaging culture requires collective effort across radiologists, radiographers, physicists, nurses, and operational staff working within safety-focused systems [29–32]. Research confirms that professional development enhances awareness, motivation, and behaviour change [33]. While most carbon emissions are produced by high-income countries (HICs) [35], LMICs such as Ghana suffer the greatest consequences of climate-driven health effects [36]. According to the World Bank, Ghana is classified as a lower-middle-income country and reflects an economy that faces persistent fiscal and infrastructural constraints despite notable progress in health and education [37].

In healthcare, sustainability involves strengthening healthcare systems to withstand emerging and future challenges [38]. The WHO defines sustainable healthcare systems as those that restore and maintain population health while minimising harm to the environment and protecting planetary resources for future generations [34]. To support this, the WHO established the “Operational framework for building climate resilient and low carbon health systems”. The goal of this framework is to increase the climate resilience of health systems to protect and improve the health of communities in an unstable and changing climate [39]. The second component of this framework, a “climate-smart health workforce,” aims to equip health workers with the skills, support, and communication capacity to address climate-related health risks and cut their own greenhouse gas emissions [39]. Additionally, the UN Framework Convention on Climate Change (UNFCCC) introduced the Action for Climate Empowerment (ACE) framework, which also promotes education, training, public awareness, participation, access to information, and international cooperation [40].

Existing research has confirmed that Ghanaian radiographers desire enhanced sustainability training [41, 42], and national policies are increasingly aligned with global climate-health mandates. However, a gap remains in connecting practitioner needs with policy frameworks. This study addresses this gap by providing the first systematic mapping of the training needs of Ghanaian radiographers to the specific competencies of the WHO’s “climate-smart health workforce” and the pillars of the UNFCCC’s Action for Climate Empowerment (ACE) framework. Although the survey was not originally designed around these global standards, applying them post-hoc provides a unique benchmarking opportunity. This approach situates the findings of the study within internationally recognised principles and creates an evidence-based and policy-aligned roadmap for developing a climate-smart radiography workforce in Ghana.

## Methods

### Study design and area

A descriptive quantitative cross-sectional study design was employed to examine the training and educational needs on sustainable radiography among 126 diagnostic radiographers in Ghana. Ghana presents a relevant setting to explore sustainable radiography, given the growing global push for climate-resilient health systems and the local challenges of implementing sustainable practices. Given these constraints, global competency frameworks from the WHO and UNFCCC ACE provide benchmarks for identifying context-specific training priorities in Ghana’s radiography workforce.

## Sample size and sampling procedure

### Sample size determination

According to the Ghana Society of Radiographers (GSR), there are approximately 320 licensed diagnostic radiographers in Ghana. The required sample size for this study was determined using Cochran’s sample size formula for a single proportion, followed by the Finite Population Correction (FPC) formula. Both formulas were applied sequentially to ensure adequate statistical power at a 95% confidence level (Z = 1.96), estimated population proportion (p) of 0.5, and a margin of error (e) of 0.05.

The first formula (Cochran’s formula for a single proportion) is given as:

$$\:{n}_{0}=\frac{{Z}^{2}\times\:p\times\:(1-p)}{{e}^{2}}$$

where,

- Z is the z-score corresponding to the desired confidence level = 1.96 for a 95% confidence level.
- *p* is the estimated proportion of the population = 0.5.
- *e* is the margin of error = 0.05.

Substituting the values:

*n*_0_=(1.96)^2^ x 0.5 x (1 − 0.5)/(0.05)^2^

*n*_0_=0.9604/0.0025

*n*_0_=384.14

Thus, the initial sample size for an infinite population n_0_ = 384.

Because the total number of licensed diagnostic radiographers in Ghana is finite (*N* = 320), the second formula, Finite Population Correction (FPC) was applied as follows:

$$\:n=\frac{{n}_{0}}{1+\frac{{n}_{0}-1}{N}}$$

Substituting the values:

*n* = 384 / 1 + (384-1)/ 320

*n* = 384 / 2.197

*n* = 174. 78

Therefore, the final required sample size for this study, after applying the finite population correction, was 175 diagnostic radiographers.

## Sampling procedure

A convenience sampling technique was then used to recruit participants. The target population comprised all licensed diagnostic radiographers licensed by the Allied Health Professions Council (AHPC) in Ghana. The study invitation and survey link were distributed via the official GSR WhatsApp platform.

### Questionnaire design and validation

The research instrument was a structured questionnaire (Supplementary File 1) developed by the research team to collect comprehensive data on sustainable radiography training needs among diagnostic radiographers in Ghana. It contained both closed-ended and open-ended questions, which were all written in English. The questionnaire covered key areas including socio-demographic and professional characteristics, familiarity with environmental sustainability, availability and scope of workplace training, perceived knowledge gaps and training preferences, and barriers to both training access and sustainable practice implementation.

To ensure content validity and clarity, the draft questionnaire was reviewed by experienced academic radiographers and revised based on their feedback. A pilot study was conducted with six practicing diagnostic radiographers in Ghana to test reliability and validity. Responses gathered from the pilot were consistent with the study objectives, thus confirming reliability of the questionnaire. Minor revisions were made based on this feedback to improve clarity and reduce ambiguity. Pilot study data were excluded from the data analysis [43]. Internal consistency testing such as Cronbach’s alpha was not conducted because the questionnaire contained a mix of single-item Likert indicators and multiple-response checklists, rather than a unidimensional multi-item scale. Cronbach’s alpha assumes that items collectively measure a single underlying construct and becomes unreliable when applied to heterogeneous or single-item measures. Using alpha in such cases could produce misleading or inflated values [44, 45]. The final version of the questionnaire was distributed electronically via Google Forms to maximise accessibility and participation.

### Framework for post-hoc interpretation

The questionnaire was developed independently of the WHO Operational framework for building climate resilient and low carbon health systems (Component 2: Climate-Smart Health Workforce) and the UNFCCC Action for Climate Empowerment (ACE) framework. These frameworks were later identified during the literature review as global standards for building a climate-resilient health workforce. Following the descriptive analysis, each survey domain was reviewed and mapped to the relevant WHO Component 2 objectives (health workforce capacity, organisational capacity development, and information/awareness/communication) and ACE elements (education, training, public awareness, public participation, and public access to information).

### Data collection

The data collection used was an online questionnaire hosted on Google Forms. The questionnaire was distributed to diagnostic radiographers, with coordination support from the GSR. Participation was entirely voluntary, and each participant was allowed to submit the survey only once. To minimise the likelihood of duplicate submissions, responses were monitored for identical timestamps, demographic profiles, and answer patterns. The Google Forms setting that restricts multiple submissions was applied, and no duplicate entries were identified. A participant information sheet, included at the start of the questionnaire, explained the purpose of the study, data confidentiality, and voluntary participation. Informed consent was obtained digitally before survey completion. The questionnaire was designed to take approximately 5 min to complete. The survey was open from December 16th, 2024, to April 6th, 2025. Periodic reminders were sent throughout the data collection period to maximise participation.

### Statistical analysis

All completed questionnaire responses were exported from Google Forms into Microsoft Excel and then transferred to SPSS (Statistical Package for the Social Sciences) Version 25 for analysis. Descriptive statistics were used to summarise the data, including frequencies and percentages for all categorical variables. Spearman’s rank-order correlation analysis was performed to explore relationships between variables such as familiarity with sustainability concepts, confidence in eco-friendly practices, and perceived knowledge gaps. All statistical analyses were conducted at a 95% confidence level, with significance set at *p* < .05. Results were presented in tabular form to ensure clarity and accessibility of key findings.

### Ethical considerations

Ethical approval for the study was obtained from the Ghana Society of Radiographers (GSR), with reference number GSR/RS/PS/FTA/002/2024. The study complied with the ethical principles outlined in the 1964 Declaration of Helsinki and its subsequent amendments. Participation in the study was entirely voluntary. Before accessing the questionnaire, participants read a brief statement explaining the study’s purpose, their role, and their rights. To proceed, they were required to indicate informed consent by selecting “Yes” on the first page of the questionnaire. No personally identifiable information was collected. All responses were fully anonymised. Data were securely stored in encrypted, password-protected files accessible only to the research team. Data privacy and participant confidentiality were strictly maintained throughout data collection.

## Results

This cross-sectional survey assessed the sustainable radiography training needs of 126 diagnostic radiographers in Ghana, which represents 72% of the calculated target sample size of 175. Participant socio-demographic characteristics, including age, gender, years of experience, and type of healthcare facility, are presented in Table 1 below. The subsequent results are mapped to the Component 2 of the WHO Operational framework and the UNFCCC Action for Climate Empowerment (ACE) pillars: Table 2 (Awareness and Understanding), Table 3 (Training Availability and Content), Table 4 (Training Priorities and Knowledge Gaps), and Table 5 (Barriers to Training and Implementation).

### Participant characteristics

The socio-demographic characteristics of the radiographers are presented in Table 1 below.

**Table 1:** Socio-demographics of participants

Age	*N*	%
18–24	13	10.3
25–34	61	48.4
35–44	30	23.8
45–54	18	14.3
55 and above	4	3.2
Total	126	100.0
**Gender**		
Male	87	69.0
Female	39	31.0
Total	126	100.0
**Years of Experience in Radiography**		
1–3 years	50	39.7
4–6 years	15	11.9
7–10 years	13	10.3
More than 10 years	47	37.3
Below one year	1	0.8
Total	126	100.0
**Type of Healthcare Facility**		
Public	81	64.3
Private	45	35.7
Total	126	100.0

The survey consisted of 126 practicing radiographers, with ages ranging from 18 to over 55 years. The majority were aged 25–34 years (48.4%, *n* = 61), followed by 35–44 years (23.8%, *n* = 30). Males comprised 69.0% (*n* = 87) of the sample, and females accounted for 31.0% (*n* = 39)

### Awareness and Understanding

The level of awareness and understanding sustainability in radiography among diagnostic radiographers are presented in Table 2, mapped to WHO Component 2, Objective 3 (Information, Awareness, and Communication) and ACE pillars on Education, Public Awareness, and Public Access to Information.

**Table 2:** Awareness and Understanding of sustainability in radiography

How familiar are you with the concept of environmental sustainability in radiography?	*N*	%
Very familiar	21	16.7
Somewhat familiar	62	49.2
Not very familiar	37	29.4
Not familiar at all	6	4.8
Total	126	100.0
**How relevant do you believe sustainability practices are to the field of radiography?**		
Extremely relevant	39	31.0
Very relevant	68	54.0
Somewhat relevant	19	15.1
Total	126	100.0
**How confident are you in your knowledge of eco-friendly practices in clinical imaging?**		
Very confident	45	35.7
Somewhat confident	53	42.1
Not very confident	25	19.8
Not confident at all	3	2.4
Total	126	100.0
**Do you currently incorporate any eco-friendly practices in your radiography work?**		
Yes, regularly	43	34.1
Yes, occasionally	58	46.0
No	25	19.8
Total	126	100.0
**How supportive is your workplace of integrating sustainability practices in radiography?**		
Very supportive	36	28.6
Somewhat supportive	37	29.4
Neutral	45	35.7
Not supportive	8	6.3
Total	126	100.0

Majority of diagnostic radiographers (49.2%, *n* = 62) reported being somewhat familiar with environmental sustainability in radiography. Regarding relevance, 54.0% (*n* = 68) considered sustainability practices very relevant to radiography. Confidence in knowledge of eco-friendly practices was moderate, with 42.1% (*n* = 53). In terms of practice, only 46.0% (*n* = 58) of radiographers occasionally incorporated eco-friendly practices, 34.1% (*n* = 43) did so regularly, and 19.8% (*n* = 25) did not. Workplace support for sustainability was mixed, with 35.7% (*n* = 45) reporting a neutral stance and 29.4% (*n* = 37) somewhat supportive. These responses are detailed in Table 2.

### Training availability and content

The availability of training and content are presented in Table 3, mapped to WHO Component 2, Objective 1 (Health Workforce Capacity) and ACE pillars on Training, Education, and Public Access to Information.

**Table 3:** Sustainability training availability and content

Have you received any prior training on sustainability in radiography?	*N*	%
Yes, through formal training (e.g., structured courses, CPD programs, workshops)	46	36.5
Yes, through informal training or workplace initiatives (e.g., on-the-job guidance, internal sessions)	30	23.8
No	50	39.7
Total	126	100.0
**Does your facility offer any training programs on sustainability in radiography?**	**N**	**%**
Yes, occasionally	45	35.7
Yes, regularly	5	4.0
Not sure	9	7.1
No	67	53.2
Total	126	100.0
**If training is available**,** what topics does it typically cover?** **(Multiple responses**, ***n* = 76 reported prior training)**	**Responses**	
	**N**	**%**
Energy-efficient practices in imaging	47	61.8%
Waste management and reduction	53	69.7%
Radiation dose optimization	76	100.0%
Eco-friendly protocols for patient care	56	73.7%
Others (No program on sustainability, no training, none, none really)	15	19.7%
**How well does the available training prepare you to implement sustainable practices?**	**N**	**%**
Moderately well	52	41.3
Not very well	24	19.0
Very well	39	31.0
Not well at all	11	8.7
Total	126	100.0
**Have you received on-the-job guidance or demonstrations on sustainability practices from colleagues or supervisors?**		
Yes	60	47.6
No	66	52.4
Total	126	100.0
**Where would you prefer to receive training on sustainability practices?**		
Professional association seminars and webinars	59	46.8
Self-Directed Learning	10	7.9
Workplace CPD sessions	37	29.4
Online courses	20	15.9
Total	126	100.0

Of the 126 radiographers, (39.7%, *n* = 50) reported no sustainability training. Over half (53.2%, *n* = 67) indicated their facility offered no sustainability training programs, while (35.7% *n* = 45) reported occasional training. The majority of respondents selected radiation dose optimisation as the most frequently covered when the concept of sustainability is being discussed, followed by eco-friendly protocols, waste management, and energy-efficient practices. Regarding training effectiveness, 41.3% (*n* = 52) rated available training as moderately well in preparing them for sustainable practices, 31.0% (*n* = 39) as very well and 8.7% (*n* = 11) as not well at all. Nearly half (47.6%, *n* = 60) had received on-the-job guidance or demonstrations on sustainability practices from colleagues or supervisors, while 52.4% (*n* = 66) had not.

### Training priorities and knowledge gaps

Table 4 presents priority areas for training and knowledge gaps among diagnostic radiographers, mapped to WHO Component 2, Objective 1 (Health Workforce Capacity) and ACE pillars on Training, Education, and Public Participation.

**Table 4:** Priority areas for training and knowledge gaps

Which areas would you like additional training on? (Multiple responses, *n* = 126)	Responses			
	*N*		%	
Developing eco-friendly protocols in radiology	92		73.0%	
Reducing radiation dose exposure	75		59.5%	
Safe disposal of radiological materials	75		59.5%	
Managing waste and recycling in imaging departments	85		67.5%	
Reducing energy consumption in radiography	95		75.4%	
**How much do you agree with the following statement: “I feel there are gaps in my knowledge about sustainability practices in radiography”**		**N**		**%**
Strongly agree		40		31.7
Agree		78		61.9
Strongly disagree		2		1.6
Disagree		6		4.8
Total		126		100.0
**Do you believe that training on sustainable practices specifically tailored to Ghana’s healthcare system would be beneficial for you?**				
Yes		120		95.2
Not sure		4		3.2
No		2		1.6
Total		126		100.0

Among the requested training areas, reducing energy consumption in radiography was selected by the majority respondents (75.4%, *n* = 95), followed by developing eco-friendly protocols in radiology (73.0%, *n* = 92), managing waste and recycling in imaging departments (67.5%, *n* = 85), and both reducing radiation dose exposure and safe disposal of radiological materials (59.5% each, *n* = 75). Almost all of the radiographers agreed or strongly agreed (93.6%, *n* = 118) with the statement, “I feel there are gaps in my knowledge about sustainability practices in radiography”.

### Barriers to training and implementation

Table 5 presents the reported barriers to training and implementation, mapped to WHO Component 2, Objective 2 (Organisational Capacity Development) and ACE pillars on Public Access to Information, Training, and Public Participation.

**Table 5:** Barriers to sustainability training and implementation

What are the main barriers to implementing sustainable practices in your facility? (Multiple responses, *n* = 126)	Responses	
	*N*	%
Insufficient time for training	53	42.1%
Lack of accessible training materials	74	58.7%
Limited knowledge about sustainability practices	79	62.7%
Limited support from management	78	61.9%
Lack of resources and funding	86	68.3%
Others	1	0.8%
**What barriers specific to your healthcare facility make sustainability implementation challenging?**		
Infrastructure limitations	64	50.8%
Shortage of trained personnel	79	62.7%
Lack of management support	72	57.1%
Limited funding or budget	80	63.5%
Others (e.g., insufficient time, none, no barriers)	64	50.8%
**How likely are you to participate in sustainability training if it were made available?**	**N**	**%**
Very likely	111	88.1
Somewhat likely	14	11.1
Not likely	1	0.8
Total	126	100.0

Among the respondents, key barriers were lack of resources/funding (68.3%), limited knowledge (62.7%) and limited management support (61.9%). Facility-specific barriers were led by limited funding (63.5%), followed by shortage of trained staff (62.7%) and infrastructure limitations (50.8%). If training were available, 88.1% were more likely to participate, compared to 0.8% who are likely not to participate

### Correlation findings

Table 6 presents Spearman’s rank-order correlations between key variables related to sustainability education.

**Table 6:** Spearman’s rank-order correlations among key variables

Variable pairs	Spearman’s rho	*P*-value	95% confidence interval level	
			Lower	Upper
Confidence level ↔ Familiarity with sustainability concepts	0.319**	***< 0.001***	0.152	0.491
Agreement with knowledge gap ↔ Familiarity with sustainability concepts	− 0.323	***< 0.001***	− 0.486	− 0.165
Confidence level ↔ Agreement with knowledge gap	− 0.202	***0.023***	− 0.370	− 0.025

**. Correlation is significant at the 0.01 level (2-tailed)

Confidence in eco-friendly practices was positively correlated with familiarity with sustainability concepts (ρ = 0.319, *p* < .001). Familiarity with sustainability concepts was negatively correlated with agreement with knowledge gaps (ρ = –0.323, *p* < .001). Confidence in eco-friendly practices was also negatively correlated with perceived knowledge gaps (ρ = –0.202, *p* = .023).

## Discussion

This study assessed awareness, training availability, priorities, and barriers to sustainable radiography among diagnostic radiographers in Ghana. The findings are interpreted using the WHO Operational Framework for Building Climate Resilient and Low Carbon Health Systems (Component 2: Climate-Smart Health Workforce) and the UNFCCC ACE pillars. The key results show that while most participants recognised sustainability as highly relevant, confidence and training opportunities were limited. Topics for training were centred on energy reduction, eco-friendly protocols, and waste management whilst barriers included lack of funding, limited management support, and insufficient knowledge. The discussion is organised thematically in line with these frameworks. It is worth noting that the estimated sample size of the study was 175, but the study received responses from 126 respondents. Using the G*Power software (v3.1.9.7) with a sample size (n) = 126 and alpha = 0.05 two-sided, post-hoc calculation yielded statistical power of 94% to detect correlations of at least an effective sample size (ρ) = 0.3. This makes the sample adequate to generalise our findings to the population.

### The awareness-confidence gap in sustainability knowledge

The study appears to reveal a disconnect between awareness and practical confidence regarding environmental sustainability, mimicking findings in previous studies. Majority of diagnostic radiographers (65.9%, as shown in Table 2) reported familiarity with the concept, and 85.0% deemed sustainability practices relevant to radiography, reiterating findings by other previous studies [41, 42]. This perceived relevance reflects the growing recognition in healthcare of its environmental footprint and the ethical imperative for sustainable practices [46]. However, this general awareness does not translate into strong practical confidence. Only 35.7% (as shown in Table 2) felt “very confident” in their knowledge of specific eco-friendly practices, while 42.1% were only somewhat confident, and a significant minority (22.2%) felt unfamiliar or not confident. This gap between theoretical understanding or perceived importance and practical knowledge may align with the WHO Component 2, Objective 3, which stresses the need for effective information systems and communication strategies to support a climate-smart health workforce. This gap is a well-documented phenomenon among diagnostic radiographers internationally [14, 41, 47]. Similarly, a study across 13 health disciplines found strong content knowledge (90.8%) but low confidence to ‘explain’ (36.9%) or ‘inspire’ students (44.2%) regarding sustainability in health education, with 67.5% unsure how best to teach it [48]. Other studies highlighted that while healthcare professionals are aware of environmental impacts, barriers like insufficient resources, lack of institutional support, and competing priorities hinder practical implementation [3, 6, 47].

Notably, the correlation analysis supports this finding. There was a significant positive correlation between familiarity with sustainability concepts and confidence in eco-friendly practices (ρ = 0.319, *p* < .001, as shown in Table 6), consistent with research showing that foundational knowledge is a prerequisite for developing practical skills and confidence [6, 14]. Conversely, familiarity with sustainability concepts and confidence in eco-friendly practices were both negatively correlated with agreement on having knowledge gaps (ρ = –0.323, *p* < .001; ρ = –0.202, *p* = .023 respectively), which may indicate that greater familiarity and higher confidence are associated with fewer perceived knowledge gaps. These findings reinforce the WHO Climate-Smart Health Workforce Competencies, which identify core knowledge and skills as essential for effective action, and the UNFCCC ACE framework, which stresses training that fosters both conceptual understanding and empowerment.

Several factors likely contributed to this gap among Ghanaian radiographers. Firstly, radiography curricula in Ghana, while providing a foundation, face challenges like a lack of harmonisation across institutions and resource constraints [49]. International calls emphasise integrating sustainability into health professional education to equip the workforce with the necessary knowledge, values, and confidence [1, 42]. This is consistent with WHO’s emphasis on building climate-smart competencies, including critical knowledge, skills, attitudes, and motivation to deliver climate-resilient, low-carbon healthcare. Yet most Ghanaian radiographers in this study appear to lack these core elements. Secondly, the lack of readily available, context-specific practical guidelines and protocols for sustainable radiography in the Ghanaian setting likely hinders the translation of general awareness into concrete actions. This may reflect gaps in ACE elements of ‘Education’, ‘Training’, and ‘Public Awareness’, which remain poorly operationalised in the current radiography context. Ghana’s inability to institutionalise these mechanisms limits radiographers’ ability to engage meaningfully in climate mitigation through practice. Additionally, the resource limitations prevalent in many LMICs [50], may limit opportunities to experiment with and implement eco-friendly practices, thereby reducing confidence.

### Deficiencies in sustainability training availability and content

The study highlights the inadequate provision of sustainability training for diagnostic radiographers in Ghana. Nearly 40% (as shown in Table 3) reported receiving no training. This finding is worrying and indicates that a substantial portion of the workforce lacks foundational knowledge in this area [41]. Furthermore, more than half of diagnostic radiographer stated their facility offers no sustainability training programs. This lack of both pre-service and in-service training is in line with international observations of insufficient sustainability education within radiography and broader healthcare curricula [1, 14, 42]. Global calls are being made to integrate Education for Sustainable Healthcare into health professional training programs to equip future professionals with the necessary knowledge, values, confidence, and capacity [1, 42]. The WHO’s framework reinforces this need, establishing that every health worker must be trained to deliver climate-resilient and low-carbon care through knowledge, values, and behaviours [39].

For the minority who reported receiving some training, the content appears narrowly focused. Radiation dose optimisation was the most frequently covered topic, followed by eco-friendly protocols, waste management and energy-efficient practices. While dose optimisation is crucial for patient safety and resource stewardship, it represents only one facet of environmental sustainability in radiology. Comprehensive sustainability frameworks encompass a broader range of high-impact areas, including energy consumption, waste management, sustainable procurement, water usage, and low-carbon infrastructure [3, 51–53]. These topics align closely with WHO’s recommended competency domains particularly sustainable practice and risk reduction yet are underrepresented in the reported training content. This mismatch limits the readiness of Ghanaian radiographers to meet global practice standards.

The limited coverage of areas like energy efficiency and comprehensive waste management in existing Ghanaian training suggests an incomplete approach that fails to address the full environmental footprint of diagnostic imaging [6, 47, 51]. Moreover, the absence of training linked to civic engagement, public awareness, or collaborative capacity building principles central to the UNFCCC ACE further weakens the systemic response to climate-health priorities in diagnostic radiography. ACE emphasises integrating sustainability into educational systems to empower professionals through access to knowledge and opportunities for participation. This is currently largely absent in radiography CPD and undergraduate training.

The perceived effectiveness of the available training is low. A majority felt it prepared them only “moderately well” or “not very well/not well at all” for practical implementation. Coupled with the finding that over half received no on-the-job guidance, this may suggest that current training efforts, where they exist, are insufficient to build practical competence and confidence. In terms of WHO’s framework, this indicates a failure to translate competency domains into measurable practice change particularly regarding leadership, communication, and climate adaptation. This inadequacy may contribute to the awareness-confidence gap discussed previously and represents a major barrier to translating the high perceived relevance of sustainability into meaningful action within Ghanaian radiography departments.

### Training priorities

The knowledge gaps perceived by Ghanaian radiographers (93.6% agreed or strongly agreed that such gaps exist) translate into clear priorities for future training, directly reflecting the major environmental impacts of their field [41]. Reducing energy consumption emerged as the top priority (as shown in Table 4), closely followed by developing eco-friendly protocols, managing waste and recycling, reducing radiation dose and safe disposal of radiological materials. This prioritisation aligns strongly with the known environmental hotspots in diagnostic imaging [3, 14]. Radiology departments are significant energy consumers, particularly due to energy-intensive modalities like CT and MRI, which can consume vast amounts of electricity, often with substantial non-productive (idle time) energy use [14, 42, 53]. Studies estimate that idle energy use can be 40–91% of total consumption for some devices [3, 47, 54]. Another major concern is waste generation, including single-use plastics, contrast media, and electronic waste from equipment disposal [15, 51]. The focus on eco-friendly protocols and safe disposal further reveals the need for practical and sustainable workflows.

Comparing these priorities with those identified internationally reveals considerable overlap, particularly with HICs, where energy reduction and waste management consistently emerge as key focus areas for sustainable radiology [42, 55]. Ghana’s status as a LMIC requires a different strategic approach, whereas HICs can invest in advanced energy-efficient equipment and comprehensive waste management infrastructure, LMICs such as Ghana must adopt cost-effective and scalable interventions that operate within existing resource limitations [50]. These may include optimising the use of existing imaging equipment, introducing low-cost waste segregation systems, and developing targeted training programmes that promote behavioural change and resource optimisation among diagnostic radiographers [14, 50].

This local specificity supports the WHO framework, which emphasises context-responsive training that adapts core domains such as ‘sustainable practice’ and ‘health system resilience’ [39]. This addresses the realities of different geographic, economic and cultural settings. The high prioritisation of energy reduction, waste management and eco-friendly protocols in this current study aligns with these domains but highlights the need for competency translation into feasible and locally achievable actions. Moreover, the expressed desire for Ghana-specific training aligns strongly with the UNFCCC ACE framework, particularly its pillars on ‘Education’, ‘Training’, and ‘Public Awareness’, which stress national relevance, accessibility, and participatory design [40]. ACE promotes training that is co-produced with practitioners and integrated into existing systems to ensure professionals have both competence and agency to act within constrained environments [40]. This reinforces the significance of that finding that an overwhelming 95.2% of respondents desire training specifically tailored to Ghana’s healthcare system. This preference stresses the need for educational content that moves beyond generic principles to address the operational challenges, available technologies, resource limitations, regulatory frameworks, and cultural settings within Ghana [56].

### Interplay of barriers to sustainability implementation and training

Ghana’s classification as a lower-middle-income country (LMIC) by the World Bank provides an important context for understanding the systemic barriers identified in this study [37]. This current study identifies interconnected barriers hindering both sustainable practice implementation and access to relevant training in Ghana. Key obstacles to implementation (as shown in Table 5) were lack of resources and funding, limited sustainability knowledge and limited management support, mirroring findings from international studies [14, 57]. Local barriers such as limited funding/budget (63.5%), shortage of trained personnel (62.7%), lack of management support, and infrastructure limitations (50.8%) show the operational constraints common in health systems of LMICs [6, 50]. Infrastructure issues, including unreliable power and inadequate waste disposal limit feasibility of certain strategies [42, 50, 58]. Staffing shortages, worsened by brain drain [19], reduce capacity for implementation and contribute to time constraints, a barrier also noted internationally [59], and in this study. Barriers to training access were similar. Limited knowledge itself (21.3%) perpetuates a cycle where lack of understanding reduces training uptake. Lack of accessible training materials (19.9%) and management support (21.0%) further limit opportunities [31]. Time constraints (42.1%) from heavy clinical workloads, compounded by staffing shortages [15, 19], and restricted access to online training courses or reliable internet, worsens the problem.

The WHO framework addresses these systemic constraints and emphasises the need for organisational, financial, and workforce enablers, including leadership for climate action and risk management [39]. The UNFCCC ACE framework likewise identifies ‘training’ and ‘public participation’ as pillars requiring structural investment [40]. Without infrastructural stability, institutional support, and resource access, ACE’s objectives cannot be realised. Ghana’s case shows that climate action is constrained not only by knowledge gaps but by broader systemic limitations. The interaction of these barriers is significant, funding limitations affect infrastructure, staffing, and training provision and knowledge gaps hinder both implementation and demand for training. Additionally, lack of leadership support reduces motivation and resourcing, and staffing shortages create time pressures that block participation [60].

These findings suggest structural limitations common in LMICs such as Ghana, where financial pressures, ageing imaging equipment, and unreliable infrastructure shape the limits of what sustainable radiography can realistically achieve. In such settings, progress depends less on high-cost technological upgrades and more on low-cost operational adaptations that can be implemented within existing resources. Practical examples include reducing MRI idle-time energy use, introducing basic waste-segregation systems, and embedding sustainability into routine clinical workflows [61, 62]. These strategies point out the need for workforce capacity, leadership engagement, and institutional support aligned with WHO and UNFCCC ACE frameworks to enable meaningful sustainability action in LMICs.

### Limitations of the study

A significant limitation is that the achieved sample size (*n* = 126) fell short of the calculated target (*n* = 175), which may reduce the statistical power of the correlational analyses. The use of convenience sampling via an online platform and WhatsApp further constrains representativeness, as radiographers without regular internet access or those not engaged in the GSR WhatsApp platform may have been excluded. This approach may overrepresent digitally connected or professionally engaged radiographers while underrepresenting those in remote or resource-limited areas. The reliance on voluntary self-selection also risks response bias, as those with a stronger interest in environmental sustainability may have been more likely to participate. Additionally, the original questionnaire was not developed using the WHO Operational Framework for Building Climate Resilient and Low Carbon Health Systems (Component 2: Climate-Smart Health Workforce) and the UNFCCC ACE frameworks as these were applied post-hoc as interpretive lenses and limits the ability to directly assess alignment with framework domains. Finally, readers are advised to interpret the reported proportions and correlations as indicative of trends rather than definitive representations of the entire radiography workforce in Ghana.

## Conclusion

Diagnostic radiographers in Ghana recognise the importance of environmental sustainability but lack the practical competence to implement it, largely due to inadequate, context-specific training and systemic resource constraints typical of LMICs. These constraints highlight the need for targeted and cost-effective sustainability training tailored to Ghana’s radiography setting and grounded in the WHO Operational Framework and UNFCCC ACE pillars.

## Supplementary Information

Below is the link to the electronic supplementary material.

Supplementary Material 1

## Data Availability

The dataset used and/or analysed during the current study is available from the corresponding author on reasonable request. The questionnaire is available as Supplementary File 1.
