# Supplementary material for: Climate-smart radiography in Ghana: training needs of diagnostic radiographers mapped to the WHO operational framework and UNFCCC Action for Climate Empowerment (ACE)
Source: BMC Health Serv Res. 2026 Jan 7;26:172. doi: 10.1186/s12913-025-13881-5 (PMC12869928; doi:10.1186/s12913-025-13881-5)
Supplement: Supplementary file 1 — Supplementary Material 1 [file 12913_2025_13881_MOESM1_ESM.pdf]

PARTICIPANT INFORMATION SHEET

Climate-Smart Radiography in Ghana: Training Needs of Diagnostic Radiographers Mapped to the WHO Operational Framework and UNFCCC Action for Climate Empowerment (ACE)

Introduction

You are invited to participate in a research study on environmental sustainability in radiography. This study aims to assess the current levels of awareness and specific training needs regarding sustainable practices among radiography professionals and students in Ghana. Your responses will help to identify potential areas for curriculum and training program development, supporting sustainable healthcare practices in radiography.

Why have I been invited?

You have been invited to participate because you are either a practicing diagnostic radiographer or a final-year radiography student in Ghana, and your insights are essential for understanding the sustainability training needs in your field.

What is the purpose of this study?

This study seeks to understand the level of awareness, current training, and specific educational needs related to environmental sustainability among radiography professionals and students in Ghana. The findings will inform recommendations for enhancing sustainability education and training in the radiography field.

What does participation involve?

If you agree to participate, you will complete an online questionnaire that will take approximately 10 minutes. The questionnaire consists of questions about your background, awareness of sustainability practices, any prior training you have received, and your views on areas where additional training may be beneficial. Your responses will be collected anonymously.

Do I have to take part?

Participation in this study is entirely voluntary. You are free to decline to participate or to withdraw at any time by simply exiting the survey without submitting your responses. No consequences will result from choosing not to participate.

Will my taking part be confidential?

Yes. The questionnaire is anonymous, and no personally identifiable information (such as your name or email) will be collected. All data will be securely stored and accessible only to the research team. Aggregated results will be reported, ensuring that individual responses cannot be identified.

What are the benefits of taking part?

While there may be no direct benefit to you, your responses will contribute to a better understanding of sustainability training needs in radiography. The findings could help shape future training programs and curriculum updates, supporting the adoption of sustainable practices within the Ghanaian healthcare system.

Are there any risks in taking part?

There are no risks associated with this study. You will only be asked to share your opinions and experiences regarding sustainability practices, and all responses are anonymous.

How will the information be used?

The data collected will be analyzed to identify trends in sustainability awareness and training needs among radiographers and students. Results may be published in academic journals or presented at conferences, but no individual participant will be identifiable.

Who has reviewed this study?

This study has been reviewed and approved by the Ethics Committee of the Ghana Society of Radiographers ensuring that it meets ethical standards for research involving human participants.

Contact for Further Information

If you have any questions or concerns about this study, please feel free to contact Emery Christian Ven via emerychristianven@gmail.com

Consent

By selecting “I Agree” at the start of the questionnaire, you indicate that you have read and understood the information provided and consent to participate in the study. You understand that

your participation is voluntary and that you may withdraw at any time by exiting the survey without submitting your responses.

Would you like to participate in this study

- Yes
- No

<u>FOR DIAGNOSTIC RADIOGRAPHERS</u>

Section A: Demographic Information

1. **Age**:
    - 18-24
    - 25-34
    - 35-44
    - 45-54
    - 55 and above
2. **Gender**:
    - Male
    - Female
    - Prefer not to say
3. **Years of Experience in Radiography**:
    - Below one year
    - 1-3 years
    - 4-6 years
    - 7-10 years
    - More than 10 years
4. **Type of Healthcare Facility**:
    - Public hospital
    - Private hospital
5. **Have you received any prior training on sustainability in radiography?**

- Yes, through formal training (e.g., structured courses, CPD programs, workshops, or seminars with certification or specific learning outcomes on sustainability in radiography)
- Yes, through informal training or workplace initiatives (e.g., on-the-job guidance, internal sessions, self-directed learning, or informal guidance on sustainable practices)
- No

Section B: Sustainability Awareness and Understanding

6. **How familiar are you with the concept of environmental sustainability in radiography?**
    - Very familiar
    - Somewhat familiar
    - Not very familiar
    - Not familiar at all
7. **How relevant do you believe sustainability practices are to the field of radiography?**
    - Extremely relevant
    - Very relevant
    - Somewhat relevant
    - Not relevant
8. **How confident are you in your knowledge of eco-friendly practices in clinical imaging?**
    - Very confident
    - Somewhat confident
    - Not very confident

- Not confident at all

9. **Do you currently incorporate any eco-friendly practices in your radiography work?**
   - Yes, regularly
   - Yes, occasionally
   - No

10. **How supportive is your workplace of integrating sustainability practices in radiography?**
    - Very supportive
    - Somewhat supportive
    - Neutral
    - Not supportive

Section C: Sustainability Training Availability and Content

11. **Does your facility offer any training programs on sustainability in radiography?**
    - Yes, regularly
    - Yes, occasionally
    - No
    - Not sure

12. **If training is available, what topics does it typically cover? (Select all that apply)**
    - Energy-efficient practices in imaging
    - Waste management and reduction
    - Radiation dose optimization
    - Eco-friendly protocols for patient care
    - Other (please specify)

13. **How well does the available training prepare you to implement sustainable practices?**
    - Very well
    - Moderately well
    - Not very well
    - Not at all
14. **Have you received on-the-job guidance or demonstrations on sustainability practices from colleagues or supervisors?**
    - Yes
    - No
15. **Where would you prefer to receive training on sustainability practices?**
    - Workplace CPD sessions
    - Online courses
    - Professional association seminars
    - Self-Directed Learning
    - Other (please specify)

Section D: Priority Areas for Training and Knowledge Gaps

16. **Which areas would you like additional training on? (Select all that apply)**
    - Reducing energy consumption in radiography
    - Managing waste and recycling in imaging departments
    - Safe disposal of radiological materials
    - Reducing radiation dose exposure
    - Developing eco-friendly protocols in radiology

- Other (please specify)

17. **How much do you agree with the following statement: "I feel there are gaps in my knowledge about sustainability practices in radiography."**
    - Strongly agree
    - Agree
    - Disagree
    - Strongly disagree
18. **Do you believe that training on sustainable practices specifically tailored to Ghana's healthcare system would be beneficial for you?**
    - Yes
    - No
    - Not sure

Section E: Barriers to Sustainability Training and Implementation

19. **What are the main barriers to implementing sustainable practices in your facility? (Select all that apply)**
    - Lack of resources and funding
    - Limited support from management
    - Insufficient time for training
    - Lack of accessible training materials
    - Limited knowledge about sustainability practices
    - Other (please specify)
20. **How likely are you to participate in sustainability training if it were made available?**
    - Very likely

- Somewhat likely
- Not likely
- Not sure

21. **What barriers specific to your healthcare facility make sustainability implementation challenging?**
    - Limited funding or budget
    - Shortage of trained personnel
    - Lack of management support
    - Infrastructure limitations
    - Other (please specify)

Section F: Suggestions for Sustainability in Radiography

22. Do you have any general suggestions or recommendations for improving sustainability practices in radiography?
